# Alkaloids from *Tetrastigma hemsleyanum* and Their Anti-Inflammatory Effects on LPS-Induced RAW264.7 Cells

**DOI:** 10.3390/molecules23061445

**Published:** 2018-06-14

**Authors:** Cai Yi Wang, Hyun-Jae Jang, Yoo Kyong Han, Xiang Dong Su, Seung Woong Lee, Mun-Chual Rho, Heng-Shan Wang, Seo Young Yang, Young Ho Kim

**Affiliations:** 1 College of Pharmacy, Chungnam National University, Daejeon 34134, Korea; wangcaiyiamy@163.com (C.Y.W.); kkoo_@naver.com (Y.K.H.); suxiangdongnicky@163.com (X.D.S.); 2 Immunoregulatory Material Research Center, Korea Research Institute of Bioscience and Biotechnology, 181 Ipsin-gil, Jeongeup-si, Jeonbuk 56212, Korea; water815@kribb.re.kr (H.-J.J.); lswdoc@kribb.re.kr (S.W.L.); rho-m@kribb.re.kr (M.-C.R.); 3 State Key Laboratory for Chemistry and Molecular Engineering of Medicinal Resources, School of Chemistry and Pharmaceutical Sciences, Guangxi Normal University, Guilin 541004, China; whengshan@163.com

**Keywords:** *Tetrastigma hemsleyanum*, alkaloid, NO production, anti-inflammatory activity

## Abstract

Alkaloids **1**–**10** were isolated from the aerial parts of *Tetrastigma hemsleyanum* (APTH) and obtained from species of the genus *Tetrastigma* for the first time. The chemical structures of the isolated compounds were identified by NMR, UV, and MS analyses. Their anti-inflammatory activities were investigated by measuring nitric oxide (NO) production in lipopolysaccharide (LPS)-induced RAW264.7 macrophages. Among all the isolates, compounds **6**, **7** and **10** showed potent inhibitory activity against LPS-stimulated NO production in RAW264.7 cells (IC_50_: 31.9, 25.2 and 6.3 μM, respectively). Furthermore, APTH and *S*-(−)-trolline (**10**) inhibited induction of inflammatory cytokines or mediators such as interleukin-1β (IL-1β) and inducible nitric oxide synthase (iNOS) via suppression of nuclear factor κB (NF-κB) translocation into the nucleus. In addition, **10** suppressed extracellular signal-regulated protein kinase 1/2 (ERK1/2) mitogen-activated protein kinase (MAPK) phosphorylation in a dose-dependent manner. These results conclusively demonstrated that compound **10** displays anti-inflammatory activity via suppression of NF-κB activation and the ERK-MAPK signaling pathway in LPS-stimulated RAW264.7 cells.

## 1. Introduction

*Tetrastigma hemsleyanum* Diels et. Gilg, belonging to the grape family Vitaceae, is an herbaceous perennial species native to China [1]. *T. hemsleyanum*, known as “Sanyeqing”, is a well-known edible plant distributed widely in China that is commonly used in folk medicine for treatment of high fever, infantile febrile convulsion, pneumonia, snake bite, and jaundice [2]. Pharmacological studies of *T. hemsleyanum* have examined its anticancer [3], liver protective, antioxidant [4], anti-inflammatory, analgesic and antipyretic activities [5]. In addition, several studies have investigated the chemical components and biological activities of *T. hemsleyanum* leaves and roots [6]. Previous studies have indicated that the phenolic constituents of the root of *T. hemsleyanum* inhibit the viability of human cancer cells [3] and that the ethyl acetate fraction (EAF) is the major contributor to the various biological activities of the plant [7]. Although *T. hemsleyanum* has long been used as a traditional Chinese medicine, little is known about its chemical composition [4,8].

Inflammation is a protective response of tissues to harmful stimuli, including injury and invading microbes. This response is a mechanism of the innate immune system to eliminate external pathogens and repair damaged tissue by activating immune cells, blood vessels, and molecular mediators [9]. Acute or chronic inflammation under pathophysiological conditions can cause various inflammatory diseases such as cancer, septic shock, diabetes, atherosclerosis, arthritis, and inflammatory bowel disease [10]. Macrophages play a pivotal role in the inflammation process by producing proinflammatory mediators such as tumor necrosis factor alpha (TNF-α), interleukin 1β (IL-1β), interleukin 6 (IL-6), and nitric oxide (NO) in addition to serving as the host defense mechanism [11,12]. Among the inflammation-related products, NO generally modulates vascular tone to control neuronal and immune functions under normal physiological conditions. However, excessive NO production leads to serious inflammatory disorders due to DNA damage, apoptosis, and reactive oxygen species (ROS) accumulation [13,14,15]. Large amounts of NO are mainly produced by inducible nitric oxide synthase (iNOS), which is regulated by gene transcription factors such as nuclear factor κB (NF-κB) and activator protein 1 (AP-1) [16]. Inhibitors of κB (IκB), which binds to inactivated NF-κB complexes and mitogen-activated protein kinases (MAPKs), are phosphorylated by other upstream signal transduction molecules such as IκB kinase (IKK) and MAPK kinases (MEKs), and the NF-κB and AP-1 transcription factors are activated in the nucleus during inflammation processes [17]. Therefore, targeting of the signaling molecules responsible for inflammation-mediated diseases is important for the development of anti-inflammation agents [18].

**Figure 1:**
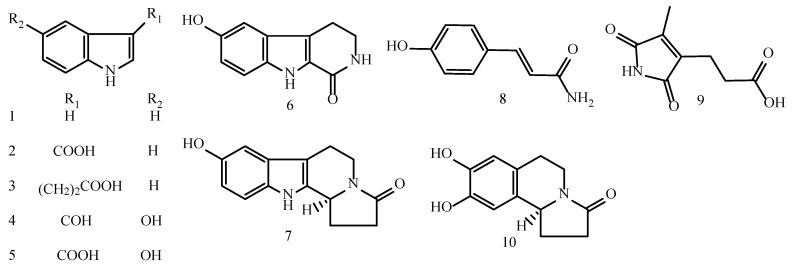
Chemical structures of the alkaloids (**1**–**10**) isolated from *T. hemsleyanum.*

In the course of our ongoing search for novel anti-inflammatory agents from important medicinal plants found in the Karst Mountains in southwest China [15], the methanol extract of the aerial parts of *T. hemsleyanum* (APTH) was shown to exhibit a considerable inhibitory effect on LPS-stimulated NO production in RAW264.7 macrophages (IC_50_: 22.69 ± 0.75 μM). In addition, we carried out a phytochemical study of APTH. Separation of the EtOAc-soluble fraction of the methanolic extract of APTH resulted in the isolation of ten alkaloids, namely, seven indole alkaloids (**1**–**7**), an amide (**8**), a maleimide (**9**), and a carboline (**10**). By comparing the spectral data of these compounds with those in the literature, the alkaloids were identified as indole (**1**) [19], indole-3-carboxylic acid (**2**) [20], indole-3-propanoic acid (**3**) [21], 5-hydroxy-indole-3-carboxaldehyde (**4**) [22], 5-hydroxy-indole-3-carboxylic acid (**5**) [22], 6-hydroxy-3,4-dihydro-1-oxo-β-carboline (**6**) [23], hippophamide (**7**) [24], 4-hydroxycinnamide (**8**) [25], pyrrole-3-propanoic acid (**9**) [26] and *S*-(−)-trolline (**10**) [27], which were isolated from the genus *Tetrastigma* for the first time (Figure 1, see Supplementary Materials). In the present paper, the isolation and structural elucidation of the alkaloids and their anti-inflammatory activities are described.

## 2. Results and Discussion

The anti-inflammatory activities of alkaloids **1**–**10** were investigated in terms of their ability to inhibit NO production in LPS-treated RAW264.7 macrophages (Table 1). Compounds **6**, **7** and **10** showed potent inhibitory activities, with IC_50_ values of 31.92 ± 0.01, 25.16 ± 0.41 and 6.28 ± 0.45 μM, respectively, and did not show cytotoxicity at the inhibitory concentration (data not shown).

Structure-activity relationship (SAR) studies of these alkaloids have shown that the lactam moiety rather than the indole group in compounds **6**, **7**, and **10** may be an important structural element for their anti-inflammatory activity. Moreover, five-membered lactam rings, such as those in compounds **7** and **10**, enhance the inhibitory effects more than six-membered lactam rings, such as that in compound **6**. Therefore, the hexahydroindolizinone pharmacophore in compound **10** may play an important role in its anti-inflammatory activity.

**Figure 2:**
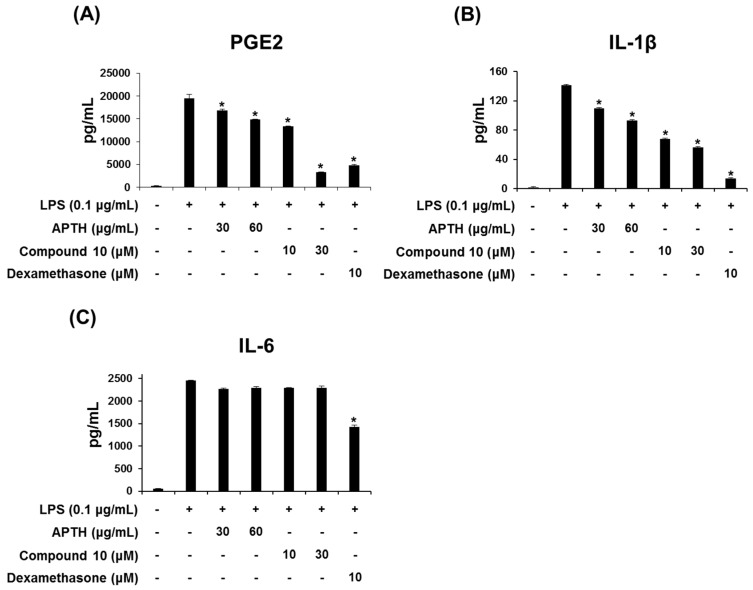
Effects of APTH and compound **10** on LPS-stimulated PGE_2_, IL-1β and IL-6 production in RAW264.7 macrophages. Cells were preincubated for 1 h with or without the test compounds and then stimulated for 16 h with LPS (0.1 μg/mL). The PGE_2_ (**A**), IL-1β (**B**), and IL-6 (**C**) levels were measured by ELISA. The results are expressed as the means ± standard error of the mean (SEM). Dexamethasone was used as the positive control. Values of * *p* < 0.05 are based on a comparison with the LPS-treated control group.

Excessive production of proinflammatory cytokines, such as TNF-α, IL-1β and IL-6, induces systemic inflammation, which can result in severe inflammatory symptoms such as acute respiratory distress syndrome (ARDS) and multiple organ dysfunction syndrome (MODS) [28]. Therefore, regulation of inflammatory cytokine levels is essential for improving the progression of acute inflammatory syndrome. To further examine the anti-inflammation activities of APTH and compound **10**, their effects on LPS-stimulated production of prostaglandin E_2_ (PGE_2_), IL-1β and IL-6 mediators were evaluated using ELISAs. As shown in Figure 2, APTH and compound **10** both reduced LPS-induced PGE_2_ and IL-1β production in a dose-dependent manner but did not affect IL-6 levels (Figure 2). PGE_2_ derived from arachidonic acid contributes to the development of various inflammatory diseases accompanied by fever, edema, and pain [29,30]. Nonsteroidal anti-inflammatory drugs (NSAIDs) are well known to exert anti-inflammatory activity through inhibition of PGHS-derived prostaglandin synthesis [31]. Therefore, the results suggest that APTH and compound **10** exhibit potent anti-inflammatory activity via inhibition of proinflammatory mediators and cytokines, such as NO, PGE_2_, and IL-1β.

**Figure 3:**
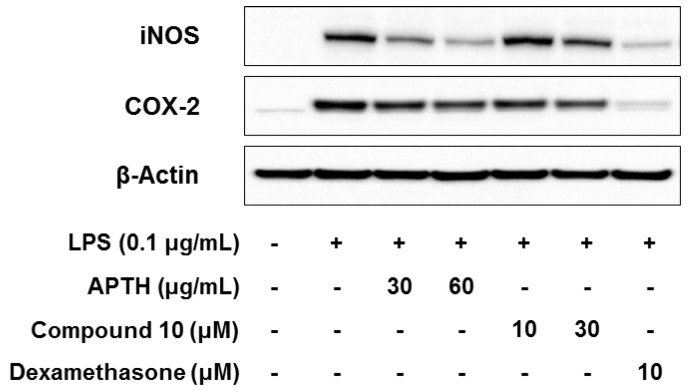
Effects of APTH and compound **10** on iNOS and COX-2 expression in LPS-induced RAW264.7 cells. Cells were pretreated for 1 h in the presence or absence of samples and then treated for 16 h with LPS (0.1 μg/mL). The iNOS and COX-2 protein expression levels were detected by Western blot analysis, and β-actin was used as the loading control. Dexamethasone served as the positive control.

We performed Western blot analyses to investigate the effects of APTH and compound **10** on iNOS and COX-2 protein expression. As shown Figure 3, both APTH and compound **10** significantly inhibited iNOS and COX-2 protein expression (Figure 3). These results indicate that APTH and compound **10** suppress the proinflammatory mediators NO and PGE_2_ through inhibition of iNOS and COX-2 protein expression and that suppression of iNOS and COX-2 expression is correlated with inhibition of NO and PGE_2_ production. In the inflammation response, NO and PGE_2_ are synthesized by iNOS and COX-2, respectively [32]. Thus, abundant iNOS and COX-2 expression promotes proinflammatory signaling pathways [33], and suppression of the expression of these inflammatory enzymes might be a therapeutic approach for the treatment of inflammation-related diseases [34].

**Figure 4:**
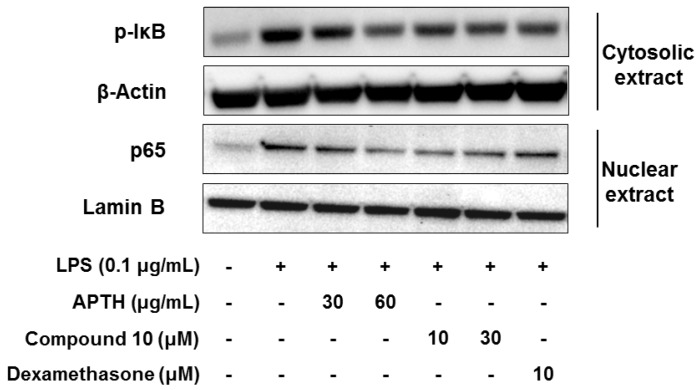
Effects of APTH and compound **10** on NF-κB signaling pathways in LPS-treated RAW264.7 macrophages. Cells were pretreated for 1 h in the presence or absence of samples and then stimulated for 1.5 h with LPS (0.1 μg/mL). Cell cytosolic and nuclear extracts were prepared to determine whether IκB phosphorylation levels and p65 translocation to the nucleus. β-Actin and Lamin B were used as loading controls. Dexamethasone served as the positive control.

The expression of inflammatory cytokines or mediators, such as NO, PGE_2_, IL-1β, IL-6, iNOS, and COX-2, is mainly regulated by activation of the NF-κB transcription factor, which plays a key role in advancing the inflammatory process [35]. Inactive complexes bound to NF-κB, the p65/p50 heterodimer, and IκB in the cytosol are degraded by phosphorylation of IκB (p-IκB), which leads to NF-κB translocation to the nucleus and transcription of proinflammatory mediators [36]. To investigate whether APTH and compound **10** affect NF-κB activation, Western blotting was performed to analyze p-IκB and p65 levels in cytosolic and nuclear extracts, respectively. As shown Figure 4, APTH and compound **10** suppressed p-IκB and translocation of p65 to a considerable degree (Figure 4). These data suggest that APTH and compound **10** decreased the NF-κB activity induced by LPS through inhibition of p-IκB in the cytosol. Therefore, the anti-inflammatory activity of APTH and compound **10** may be attributed to regulation of NF-κB signaling cascades.

**Figure 5:**
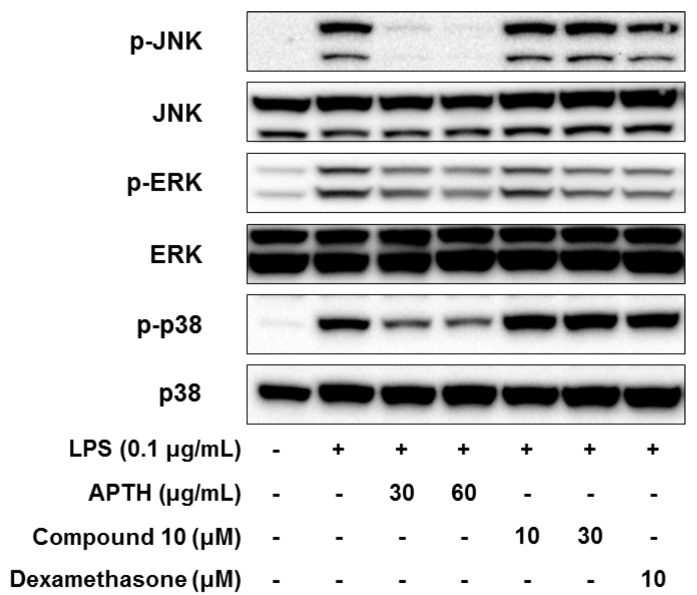
Effects of APTH and compound **10** on JNK, ERK, and p38 MAPK phosphorylation in LPS-stimulated RAW264.7 cells. Cells were pretreated for 1 h in the presence or absence of samples and then treated for 0.5 h with LPS (0.1 μg/mL). The phosphorylation of the JNK, ERK, and p38 proteins was measured by Western blotting, and total JNK, ERK, and p38 protein levels were used as loading controls. Dexamethasone served as the positive control.

MAPK is also a key mediator in promoting the inflammatory response [37]. c-Jun N-terminal kinase (JNK), extracellular signal-regulated kinase (ERK), and p38 are representative components of the MAPK signaling cascades, and their phosphorylation results in activation of proinflammatory transcription factors, such as AP-1 and cAMP response element-binding protein (CREB) [10]. Therefore, the inhibitory effects of APTH and compound **10** on LPS-stimulated MAPK activation were assessed by analyzing the phosphorylation levels of JNK, ERK, and p38 (p-JNK, p-ERK, and p-p38) using Western blotting. As shown Figure 5, APTH dose-dependently inhibited LPS-stimulated p-JNK, p-ERK, and p-p38; however, compound **10** only suppressed p-ERK (Figure 5). These findings suggest that the three major functions of MAPKs were substantially reduced by APTH, and compound **10** is involved in suppression of ERK-MAPK activation.

Similarly, a previous study demonstrated that flavonoids obtained from the roots of *T. hemsleyanum* regulate the LPS-induced inflammatory response in RAW264.7 cells via the NF-κB and JNK-MAPK signaling pathways [37]. However, the present study found that APTH can also be considered an effective anti-inflammatory agent, similar to the root extract of *T. hemsleyanum*.

Dexamethasone is a steroidal anti-inflammatory agent for treatment of inflammatory and autoimmune diseases, such as asthma, osteoarthritis, and inflammatory bowel disease [38,39,40,41,42]. Recent studies have reported drug delivery strategies to improve agent effectiveness in regulating immune responses in macrophages and microfold (M) cells [40,41,42]. Among many delivery strategies, such as nanoparticles, liposomal drug carriers, and microparticle-based drug delivery, poly(_DL_-lactic acid) microspheres containing dexamethasone demonstrated significantly higher efficacy than treatment with dexamethasone alone [42]. Therefore, employment of a drug delivery system targeting immune-related cells may be useful for improving the efficacy of small-molecule natural products such as compound **10**.

## 3. Materials and Methods

### 3.1. General Information

Optical rotations were determined on a JASCO P-2000 polarimeter (Hachioji, Tokyo, Japan). CD spectra were recorded with a Chirascan spectropolarimeter (Applied Photophysics, Leatherhead, UK). High-resolution electrospray ionization mass spectrometry (ESI-MS) analysis was conducted on an Agilent 6530 Accurate-Mass Q-TOF LC/MS system (Agilent Technologies, Palo Alto, CA, USA). NMR spectra were recorded on Bruker AM600 FT-NMR and Bruker BioSpin 400 NMR spectrometers (Rheinstetten, Germany). Column chromatography (CC) was performed on silica gel (Kiesel gel 60, 70–230 mesh and 230–400 mesh, Merck, Darmstadt, Germany), YMC*GEL (ODS-A, 12 nm S-150 μm, YMC, Kyoto, Japan) and Sephadex LH-20 (Sigma-Aldrich, St. Louis, MO, USA) resins. Precoated silica gel 60 F_254_ (1.05554.0001, Merck, Darmstadt, Germany) and RP-18 F_254S_ plates (1.15685.0001, Merck) were used for TLC. Preparative high-performance liquid chromatography (HPLC) separations were carried out using a Shimadzu LC-6AD (Shimadzu, Kyoto, Japan) instrument with a YMC-Pack ODS-A column (20 mm I.D. × 250 mm, S-5 μm) and an SPD-20A wavelength detector set at 210 nm.

### 3.2. Plant Material

The aerial parts of *T. hemsleyanum* were collected from Linchuan County, Guilin City, Guangxi Zhuang Autonomous Region in July 2016. The plant was identified by Professor Shao-Qing Tang (Guangxi Normal University), and a voucher specimen (No. 20160110) was deposited at the School of Life Sciences, Guangxi Normal University.

### 3.3. Extraction and Isolation

The dried stems and leaves of *T. hemsleyanum* (25.0 kg) were extracted with 90% ethanol three times (75 °C, 3 h each time). All the filtrates were concentrated to afford 1 kg of crude extract. The crude extract was suspended in water and then sequentially partitioned into *n*-hexane, ethyl acetate and *n*-butanol (three times each). The EAF was subjected to silica gel CC with a CH_2_Cl_2_-MeOH gradient with increasing polarity (30:1, 20:1, 15:1, 10:1, 8:1, 7:1, 6:1, 5:1, 3:1, 1:1, and 0:1), yielding four fractions (frs. 1-4). Fr. 1 was separated by silica gel CC (EtOAc-Hex, 1:2) and provided three fractions (frs. 1-1 to 1-3). Fr. 1-1 was purified by LH-20 CC (MeOH) and then subjected to HPLC (MeOH-H_2_O) to yield compound **10** (4.4 mg). Fr. 1-2 was subjected to LH-20 CC (MeOH) to give compound **2** (1.1 mg). Fr. 1-3 was also purified by LH-20 CC (MeOH) and then subjected to HPLC (MeOH-H_2_O) to give compound **6** (10.6 mg). Fr. 2 was subjected to RP-18 CC (30%, 50%, 70%, and 100% MeOH) and then to LH-20 CC (MeOH) and HPLC (MeOH-H_2_O) to afford compound **3** (9.0 mg). Fr. 3 was purified on an RP-18 column (30%, 50%, 70%, and 100% MeOH), followed by subsequent purification via LH-20 CC (MeOH) and HPLC (CH_3_CN, 0.1% TFA in H_2_O), affording compound **1** (4.5 mg). Fr. 4 was loaded onto a silica gel column by VLC and successively eluted with CH_2_Cl_2_:MeOH:H_2_O (8:1:0.125 and 8:2:0.25) to yield Fr. 4-1. Fr. 4-1 was subjected to LH-20 CC (MeOH) to obtain four sub-fractions (frs. 4-1-1 to 4-1-4). Fr. 4-1-1 was fractionated by HPLC (CH_3_CN, 0.1% TFA in H_2_O) to afford compound **7** (12.4 mg). Fr. 4-1-2 was separated by HPLC (CH_3_CN, 0.1% TFA in H_2_O) to yield **8** (7.0 mg). Fr. 4-1-3 was also separated by HPLC (CH_3_CN, 0.1% TFA in H_2_O) and provided compounds **4** (6.6 mg) and **5** (5.4 mg). Fr. 4-1-4 was purified by HPLC (CH_3_CN, 0.1% TFA in H_2_O) to give compound **9** (6.2 mg).

### 3.4. Measurement of NO, PGE_2_, IL-1β and IL-6 Production and Cell Cytotoxicity

RAW264.7 cells (TIB-71, ATCC, Manassas, VA, USA) were obtained from American Type Culture Collection (ATCC). NO production was measured using a previously described method [43,44]. Briefly, RAW264.7 macrophages were seeded into 96-well plates at a density of 5 × 10^5^ cells/well, and after being incubated with samples for 1 h, the cells were treated with LPS (0.1 µg/mL) for 24 h. Equal volumes of the cell culture supernatant and Griess reagent were mixed, and the absorbance of the mixtures was recorded at 550 nm. The levels of PGE_2_, IL-1β and IL-6 cytokines were measured with ELISAs. RAW264.7 cells (2 × 10^6^ cells/well) were cultured in a 6-well plate for 24 h. The cells were activated by LPS (0.1 µg/mL) for 16 h after being pretreated with samples for 1 h, and the concentrations of PGE_2_, IL-1β and IL-6 in the supernatants were measured using an ELISA kit (R&D Systems, Minneapolis, MN, USA). The cell viabilities were quantified with an MTT (3-(4,5-dimethylthiazol-2-yl)-2,5 diphenyl tetrazolium bromide) assay after 24 h of incubation with the samples [45].

### 3.5. Western Blotting Analysis

Western blot analyses were carried out as described previously [45]. In brief, RAW264.7 cells (2 × 10^6^ cell/well) were pretreated with the methanol extracts of the aerial parts of *T. hemsleyanum* (APTH) and compound **10** for 1 h, and then cells were stimulated with LPS (0.1 µg/mL) for 0.5, 1.5, and 24 h. The cytosol and nuclear fractions were extracted using an NE-PER Nuclear and Cytoplasmic Extraction Kit (Thermo Fisher Scientific, San Jose, CA, USA) according to the manufacturer’s protocol. Each cell lysate was loaded on an 8% or 10% sodium dodecyl sulfate (SDS) polyacrylamide gel, and then, the proteins were transferred onto PVDF membranes. After blocking the membrane in 0.01% Tween 20 (TBST) containing 5% skimmed milk powder for 1 h, the membrane was incubated with the appropriate primary antibody (targeting iNOS, COX-2, β-actin, p-IκB, p65, Lamin B, p-JNK, JNK, p-ERK1/2, ERK1/2, p-p38, or p38; Cell Signaling Technology, Beverly, MA, USA) at 1:1000 dilution overnight at 4 °C. The membrane was washed in TBST and incubated with the appropriate horseradish peroxide-conjugated secondary antibody at 1:2000 dilution for 30 min. After the membrane was washed in TBST, the immunoreactive bands were visualized using a West-ZOL Plus kit (iNtRON Biotechnology, Seoul, Korea) and detected with a ChemiDoc™ XRS system (Bio-Rad Laboratories Inc., Hercules, CA, USA).

### 3.6. Statistical Analysis

The data are presented as the means ± standard error of the mean (SEM). All statistical analyses were performed using Prism 5 software (GraphPad software, San Diego, CA, USA). Student’s *t*-test was used to determine significant differences between the control and experimental groups, with *p* < 0.05 indicating a significant difference.

## 4. Conclusions

In summary, ten alkaloids were isolated from APTH, and their chemical structures were determined from based on NMR and MS spectroscopic data. APTH and *S*-(−)-trolline (**10**) showed potent anti-inflammatory activity in LPS-treated RAW264.7 cells by inhibiting various inflammatory cytokines or mediators, such as NO, PGE_2_, IL-1β, iNOS, and COX-2, and these inhibitory activities are closely related to regulation of the NF-κB signaling pathway. The crude extract APTH attenuated the phosphorylation of three major MAPKs (JNK, ERK, and p38), whereas compound **10** inhibited only the activity of ERK-MAPK. Therefore, *S*-(−)-trolline (**10**) may be primarily responsible for the anti-inflammatory activity of APTH. These results revealed that APTH and *S*-(−)-trolline (**10**) could be used to develop novel therapeutic agents for treatment of inflammatory diseases.

## Figures and Tables

**Table 1:** Inhibition of NO production in RAW264.7 macrophages by alkaloids **1**–**10** ^a^.

Compounds	IC_50_ (µM)	Compounds	IC_50_ (µM)
**1**	>50	**6**	31.92 ± 0.01 *
**2**	>50	**7**	25.16 ± 0.41 *
**3**	>50	**8**	>50
**4**	>50	**9**	>50
**5**	>50	**10**	6.28 ± 0.45 *
		**Dexamethasone** ^b^	0.009 ± 0.001 *

^a^ The inhibitory activities are presented as the concentration (μM) giving 50% inhibition (IC_50_) relative to the vehicle control, and the results are the mean of three replications. ^b^ Positive control. The data are presented as the mean ± SEM (*n* = 3) of three independent experiments. * *p* < 0.05 vs. the LPS-treated control group.

## Supplementary Materials

The following are available online. Physical and spectroscopic data of compounds **1**–**10**.
