# Supplementary material for: Alkaloids from Tetrastigma hemsleyanum and Their Anti-Inflammatory Effects on LPS-Induced RAW264.7 Cells
Source: Molecules. 2018 Jun 14;23(6):1445. doi: 10.3390/molecules23061445 (PMC6099609; doi:10.3390/molecules23061445)
Supplement: Supplementary file 1 [file molecules-23-01445-s001.pdf]

Supplementary Materials: Alkaloids from *Tetrastigma hemsleyanum* and Their Anti-Inflammatory Effects on LPS-Induced RAW264.7 Cells

Cai Yi Wang [1,†], Hyun-Jae Jang [2,†], Yoo Kyong Han [1], Xiang Dong Su [1], Seung Woong Lee [2], Mun-Chual Rho [2], Heng-Shan Wang [3], Seo Young Yang [1,*] and Young Ho Kim [1,*]

Physical and Spectroscopic Data of Compounds

*1. Indole (**1**)*

- C_8H_7N, colorless solid; ^{1}H-NMR (400 MHz, CD_3OD) δ 8.35 (d, *J* = 8.3 Hz, 1H, H-2), 8.29 (dd, *J* = 7.0, 2.0 Hz, 1H, H-4), 7.87 (dd, *J* = 8.0, 7.3 Hz, 1H, H-5), 7.77 (d, *J* = 8.3 Hz, 1H, H-3), 7.59 (dd, *J* =8.0, 7.3 Hz, 1H, H-6), 6.68 (dd, *J* = 7.0, 2.0 Hz, 1H, H-7). ^{13}C-NMR (100 MHz, CD_3OD) δ 136.21 (C-8), 123.68 (C-2), 127.61 (C-9), 121.49 (C-5), 119.86 (C-4), 118.78 (C-6), 112.97 (C-7), 101.48 (C-3).

*2. Indole-3-carboxylic acid (**2**)*

- $C_9H_7NO_2$, yellow solid; ^{1}H-NMR (400 MHz, CD_3OD) δ 7.95 (s, 1H, H-2), 8.07 (dd, *J* = 6.8, 2.0 Hz, 1H, H-4), 7.87 (m, 7.26–7.12, 1H, H-5), 7.59 (m, 7.26–7.12, 1H, H-6), 7.44 (dd, *J* = 6.8, 2.0 Hz, 1H, H-7). ^{13}C-NMR (100 MHz, CD_3OD) δ 169.27 (C-**C**OOH), 138.21 (C-8), 133.36 (C-2), 127.58 (C-9), 123.57 (C-5), 122.36 (C-6), 122.04 (C-4), 112.87 (C-7), 108.80 (C-3).

*3. Indole-3-propanoic acid (**3**)*

- $C_{11}H_{11}NO_3$, colorless needles; ^{1}H-NMR (400 MHz, CD_3OD) δ 7.77 (d, *J* = 8.3 Hz, 1H, H-3), 7.47 (d, *J* = 7.9 Hz, 1H, H-4), 7.27 (d, *J* = 8.1 Hz, 1H, H-7), 7.07 (s, 1H, H-2), 7.03 (t, *J* = 7.4 Hz, 1H, H-6), 6.95 (t, *J* = 7.4 Hz, 1H, H-5), 3.13 (t, *J* = 7.0 Hz, 1H, H-α), 3.02 (t, *J* = 7.0 Hz, 1H, H-β). ^{13}C-NMR (100 MHz, CD_3OD) δ 177.6 (C-**C**OOH), 138.30 (C-8), 128.13 (C-9), 124.29 (C-2), 122.74 (C-5), 120.03 (C-4), 118.86 (C-6), 112.54 (C-7), 110.25 (C-3), 41.22 (C-(**C**H_2)$_2$), 24.49 (C-(**C**H_2)$_2$).

*4. 5-Hydroxy-indole-3-aldehyde (**4**)*

- $C_9H_7NO_2$, colorless needles; UV (MeOH) λ_{max} 212, 253, 301 nm; ^{1}H-NMR (400 MHz, CD_3OD) ^{1}H-NMR (400 MHz, CD_3OD) δ 8.02 (s, 1H, H-2), 7.59 (d, *J* = 2.3 Hz, 1H, H-4), 7.32 (d, *J* = 8.7 Hz, 1H, H-7), 6.82 (dd, *J* = 8.7, 2.3 Hz, 1H, H-6).

4.1. X-ray Diffraction Analysis of Compound **4**

Single crystal of compound **4** growth from solution of methanol. The X-ray crystallographic data of compound **4** were collected on a Bruker APEX-II CCD diffractometer using Cu Kα radiation. The structures were solved bydirect methods using SHELXS-97 [1] and refined with full-matrix leastsquares calculations on F^2 using SHELXL-97 [1] via OLEX2. [2] All nonhydrogen atoms were refined anisotropically. The hydrogen atompositions were geometrically idealized and allowed to ride on theirparent atoms. Crystallographic data for **4** have beendeposited in the Cambridge Crystallographic Data Centre (CCDC).Copies of these data can be obtained free of charge from the CCDC,12 Union Road, Cambridge CB2 1EZ, UK [fax: +44 (0) 1223-336033or e-mail: deposit@ccdc.cam.ac.uk].

4.2. Crystallographic Data for **4**

A suitable crystal (0.18 × 0.15 × 0.12) was use for analysis. The data were measured using a Bruker APEX-II CCD diffractometer, using Cu Kα graphite-monochromated radiation (λ = 1.54184Å). Crystal data: $C_9H_7NO_2$, M = 161.16, orthorhombic, a = 14.5715(4) Å, b = 5.87719(15) Å, c = 8.4032(2) Å, α= 90°, β = 90°, γ = 90°, V = 719.65(3) Å^3, T = 100.01(10) K, space groupPca2$_1$, Z = 4, μ(Cu Kα) = 0.886 mm^{-1}, Dc = 1.487 g/cm^3,F(000) = 336.0. A total of 4321 reflection were collected in the range 12.148°< θ < 147.238° with1390 independent reflections [R_{int} = 0.0195, R_{sigma} = 0.0156]. The final R_1 values were0.0320 [I > 2σ(I)]. The final $wR(F^2)$ values were 0.0844 [I > 2σ(I)].The final R_1 values were 0.0322 (all data). The final $wR(F^2)$ values were 0.0850 (all data). The goodness of fit on F^2 was 1.046. Flackparameter: 0.00(10)/0.11(7). CCDC1841613 contains the supplementary crystallographic data for the structure of **4**.

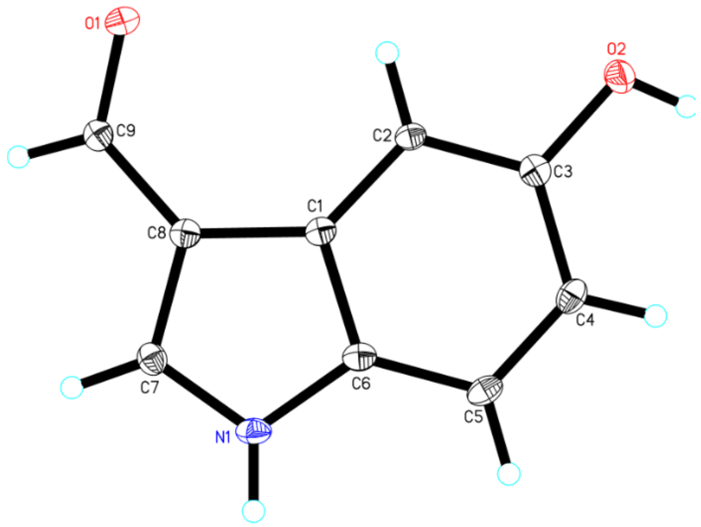

Figure S1. Single-crystal structure of compound **4**.

*5. 5-Hydroxy-indole-3-carboxylic acid (**5**)*

- $C_{10}H_7NO_3$, yellow needles; ^{1}H-NMR (400 MHz, CD_3OD) δ 8.33 (s, 1H, H-2), 7.68 (d, J = 2.4 Hz, 1H, H-4), 7.31 (d, J = 8.7 Hz, 1H, H-7), 6.81 (dd, J = 8.7, 2.4 Hz, 1H, H-6). ^{13}C-NMR (100 MHz, CD_3OD) δ 168.01 (C-**C**OOH), 150.71 (C-5), 138.08 (C-2), 135.74 (C-8), 124.76 (C-9), 113.32 (C-7), 112.47 (C-6), 106.21 (C-3), 104.21 (C-4).

*6. 6-Hydroxy-3,4-dihydro-1-oxo-β-carboline (**6**)*

- $C_{11}H_{10}N_2O_2$, amorphous powder; ESI-MS *m/z*: 203.07 $[M+H]^+$; UV (MeOH) λ_{max} 208, 306 nm; ^{1}H-NMR (400 MHz, CD_3OD) δ 7.27 (d, *J* = 8.8 Hz, 1H, H-8), 6.91 (d, *J* = 2.0 Hz, 1H, H-5), 6.84 (dd, *J* = 8.8, 2.3 Hz, 1H, H-7), 3.62 (t, *J* = 7.1 Hz, 2H, H-3), 2.94 (t, *J* = 7.1 Hz, 2H, H-4). ^{13}C-NMR (100 MHz, CD_3OD) δ 163.7 (C-1), 151.2 (C-6), 133.3 (C-8a), 127.0 (C-9a), 126.1 (C-4b), 119.3 (C-4a), 115.9 (C-7), 113.1 (C-8), 103.3 (C-5), 41.8 (C-3), 20.6 (C-4).

*7. Hippo-phamide (**7**)*

- $C_{14}H_{14}N_2O_2$, amorphous solid; ESI-MS *m/z*: 243.14 $[M + H]^+$; UV (MeOH) λ_{max} 202, 221, 277 nm; ^{1}H-NMR (400 MHz, CD_3OD) δ 7.13 (d, *J* = 8.6 Hz, 1H, H-12), 6.80 (d, *J* = 2.2 Hz, 1H, H-9), 6.65 (dd, *J* = 8.6, 2.4 Hz, 1H, H-11), 5.01 – 4.94 (t-like, *J* = 6.9 Hz, 1H, H-3), 4.45 – 4.35 (m, 1H, H-5a), 3.13 – 3.01 (m, 1H, H-5b), 2.76–2.69 (m, 2H, H-6), 2.68–2.62 (m, 1H, H-15a), 2.62–2.57 (m, 1H, H-14a), 2.49–2.38 (m, 1H, H-15b), 1.97 – 1.82 (m, 1H, H-14b). ^{13}C NMR (100 MHz, CD_3OD) δ 176.0 (C-16), 151.5 (C-10), 135.6 (C-2), 133.0 (C-13), 128.8 (C-8), 112.5 (C-12), 112.3 (C-11), 107.0 (C-7), 103.4 (C-9), 56.4 (C-3), 39.0 (C-5), 32.6 (C-15), 26.8 (C-14), 22.0 (C-6).

*8. 4-Hydroxycinnamide (**8**)*

- $C_9H_9NO_2$, amorphous powder; ESI-MS *m/z*: 162.06 $[M - H]^-$; UV (MeOH) λ_{max} 218, 328, nm; ^{1}H-NMR (400 MHz, DMSO): δ 7.50 (d, *J* = 8.5 Hz, H-2, 2H, H-6), 7.46 (d, *J* = 16.0 Hz, 1H, H-7), 6.78 (d, *J* = 8.5 Hz, 2H, H-3, H-5), 6.29 (d, *J* = 16.0 Hz, 1H, H-8). ^{13}C-NMR (150 MHz, DMSO) δ 168.2 (C-9), 159.6 (C-4), 143.7 (C-7), 131.9 (C-1), 130.0 (C-2,6), 115.8 (C-3,5), 114.8 (C-8).

*9. Pyrrole-3-propanoic acid (**9**)*

- $C_8H_9NO_4$, colorless solid; ^{1}H-NMR (400 MHz, DMSO) δ 2.53–2.50 (m, 4H, H-6, 7), 1.78 (s, 3H, H-8). ^{13}C-NMR (100 MHz, DMSO) δ 176.8 (C-COOH), 173.3 (C-2), 173.0 (C-5), 139.6 (C-3), 138.2 (C-4), 31.9 (C-CH_2COOH), 18.9 (C-CH_2CH_2COOH), 8.2 (C-CH_3).

*10. S-(−)-trolline (**10**)*

- $C_{12}H_{13}NO_3$, colorless solid; ESI-MS *m/z*: 218.05 $[M - H]^-$; UV (MeOH) λ_{max} 209, 286 nm; ^{1}H-NMR (400 MHz, CD_3OD) δ 6.36 (s, 1H, H-10), 6.35 (s, 1H, H-7), 4.56 (t, *J* = 8.0, 8.0 Hz, 1H, H-10′′), 3.93–3.83 (m, 1H, H-5a), 2.88–2.78 (m, 1H, H-5b), 2.56 (m, 1H, H-1a), 2.46–2.38 (m, 2H, H-6), 2.40–2.29 (m, 1H, H-2a), 2.25–2.15 (m, 1H, H-2b), 1.68–1.54 (m, 1H, H-1b). ^{13}C-NMR (100 MHz, CD_3OD) δ 174.3 (C-3), 143.9 (C-9), 143.8 (C-8), 128.3 (C-10′), 124.3 (C-6′), 115.0 (C-7), 111.1 (C-10), 56.8 (C-10′′), 37.3 (C-5), 31.4 (C-2), 27.4 (C-6), 27.2 (C-1).

References

1. Sheldrick, G.M. A short history of shelx. *Acta Cryst. A.* **2008**, *64*, 112–122.
2. O.V.; Bourhis, L.J.; Gildea, R.J.; Howard, J.A.; Puschmann, H. Olex2: A complete structure solution, refinement and analysis program. *J. Appl. Cryst.* **2009**, *42*, 339–341.
